# Presence of tumor-infiltrating CD8^+^ T cells and macrophages correlates to longer overall survival in patients undergoing isolated hepatic perfusion for uveal melanoma liver metastasis

**DOI:** 10.1080/2162402X.2020.1854519

**Published:** 2020-12-10

**Authors:** Junko Johansson, Jan Siarov, Roberta Kiffin, Johan Mölne, Jan Mattsson, Peter Naredi, Roger Olofsson Bagge, Anna Martner, Per Lindnér

**Affiliations:** a Department of Surgery, Institute of Clinical Sciences, Sahlgrenska Academy, University of Gothenburg, Gothenburg, Sweden; b Department of Surgery, Sahlgrenska University Hospital, Gothenburg, Sweden; c Wallenberg Centre for Molecular and Translational Medicine, University of Gothenburg, Gothenburg, Sweden; d Department of Laboratory Medicine, Institute of Biomedicine, Sahlgrenska Academy, University of Gothenburg, Gothenburg, Sweden; e Department of Infectious Diseases, Institute of Biomedicine, Sahlgrenska Academy, University of Gothenburg, Gothenburg, Sweden; f TIMM Laboratory, Sahlgrenska Center for Cancer Research, Sahlgrenska Academy, University of Gothenburg, Gothenburg, Sweden; g Transplant Institute, Sahlgrenska University Hospital, Gothenburg, Sweden

**Keywords:** Uveal melanoma, isolated hepatic perfusion, T cells, CD8+, CD68+

## Abstract

Uveal melanoma is a malignant tumor of the eye that often metastasizes to the liver conferring poor prognosis. When comparing immune profiles in peripheral blood of untreated patients with uveal melanoma liver metastasis and healthy blood donors, it was observed that immune cells of uveal melanoma patients carried immunosuppressive features. Patient blood contained an increased content of CD14^+^HLA-DR^−/low^ M-MDSCs and inflammatory CD16^+^ monocytes, while their dendritic cells expressed lower levels of activation markers. Melanoma patients also harbored an enhanced fraction of CD4^+^Foxp3^+^ regulatory T cells, while their effector T cells expressed lower levels of the activation marker HLA-DR. Biopsies from liver metastases were obtained from patients with uveal melanoma that subsequently underwent hyperthermic isolated hepatic perfusion (IHP) with melphalan. There were trends indicating a positive correlation between a high infiltration of CD8^+^ T cells in metastases and an activated immune cell profile in blood. High metastatic infiltration of CD8^+^ T cells and CD68^+^ macrophages, but not of immunosuppressive CD163^+^ macrophages, correlated to a longer overall survival in patients treated with IHP. Hence, while the immune system of patients with uveal melanoma shows signs of immunosuppression, the presence of activated immune cells may correlate to a longer survival, at least following IHP treatment.

## Introduction

Uveal melanoma is a rare form of melanoma that arises in the uvea of the eye. Though it constitutes only a small fraction of all melanomas, it is the most common intraocular tumor in adults^1,2^. After five years, one fourth of all patients have developed metastatic disease, the majority with metastases in the liver.^3,4^ The 1-year overall survival (OS) rate after diagnosis of metastatic disease is 20% and the 2-year OS rate is only 4–8%.^3,4^ Despite a battery of different treatment strategies having been tested, none has yet proven to increase overall survival.^5^

Uveal melanoma is known for having a low immunogenicity and treatment with modern immunotherapy such as anti-CTLA-4 and anti-PD1 checkpoint inhibitors have limited response rates.^6,7^ In order to evaluate if there is a rationale for developing other strategies of immunotherapies in metastatic uveal melanoma, it is of importance to fully characterize the immune profiles in patients with this disease. To this end, we have characterized immune cells in both peripheral blood and in metastatic liver biopsies from uveal melanoma patients prior to treatment with isolated hepatic perfusion with melphalan.

Isolated hepatic perfusion (IHP) with the alkylating agent melphalan is a treatment option for patients with isolated liver metastases. IHP is a locoregional treatment in which it is possible to achieve very high local concentrations of a chemotherapeutic agent compared to systemic administration. During IHP the liver is surgically isolated from the systemic circulation and connected to a heart-lung machine, and high doses of melphalan are perfused through the liver at mild hyperthermia (40°C) during one hour. The response rate after IHP is approximately 70%,^8^ and a potential survival benefit for IHP patients is currently being investigated in a phase III randomized clinical trial (the SCANDIUM trial).^9^

We and others have previously shown that isolated limb perfusion (ILP) with melphalan to patients with *in situ* metastatic cutaneous melanoma stimulates the immune system, and that patients with activated T cells before ILP achieve a better response to the treatment.^10–14^ This prompted us to investigate if IHP may similarly rely on the immune system of the patients for optimal clinical effect. To test this hypothesis, we correlated the presence of CD8^+^ T cells and CD68^+^ macrophages in liver metastases obtained prior to IHP with survival of the patients.

## Materials and methods

### Patients and IHP

Patients with uveal melanoma liver metastasis were recruited from two different cohorts, all undergoing IHP at the Sahlgrenska University Hospital in Gothenburg, Sweden. The first cohort included patients from the SCANDIUM trial (ClinicalTrials.gov identifier number: NCT01785316) with sampling of both peripheral blood and biopsies from liver metastases. For detailed information about the study protocol and the enrollment criteria, see.^9^ The second cohort contained liver metastasis biopsies from patients treated with IHP before the initiation of the SCANDIUM trial. Detailed information about all patients can be found in Table 1. All samples were obtained prior to IHP. Survival data were available for patients in the second cohort. None of the patients in this cohort received treatment with anti-PD-1 checkpoint inhibitors following IHP. All patients were followed until time of death, and median follow-up time was 18 months. All patients gave written consent and the study was approved by the Regional Ethical Review Board in Gothenburg, Sweden (No. 144–13).

**Table 1.** Patient characteristics

	Cohort 1	Cohort 2
Sex		
Female	16 (57%)	10 (71%)
Male	12 (43%)	4 (29%)
Age (median + range)	65 years (34–80)	60 years (27–76)
Primary treatment		
Enucleation	11 (44%)	6 (46%)
Radiation	14 (56%)	6 (46%)
Both	–	1 (8%)
Time between primary treatment and IHP (median + range)	24 months (3–93)	14 months (10–56)

During IHP catheters are inserted into the iliac vein and the axillary vein to allow for shunting of blood from the lower extremity. A catheter is placed in the retrohepatic portion of the caval vein for perfusion outflow and the caval vein is clamped suprahepatic and below the catheter. The portal vein is clamped and the proper hepatic artery is cannulated via the gastroduodenal artery. The catheters are then connected to the perfusion system. When steady-state conditions in the perfusion circuit are established, melphalan (1 mg/kg bodyweight) is added to the perfusion system. The perfusion is performed with a target liver temperature of 40°C. The leakage from the perfusion circuit is continuously recorded. Perfusion is continued for 60 minutes, after which the perfusion is discontinued and the liver is irrigated. The shunts and the perfusion circuit are disconnected and the catheters are removed.

### Purification of PBMCs

Peripheral blood mononuclear cells (PBMCs) from patient blood were obtained from BD Vacutainer® CPT™ cell preparation tubes (BD Biosciences, #362782) according to the manufacturer’s protocol. Control PBMCs were purified from buffy coats from anonymous healthy donors obtained from the Component Laboratory at Sahlgrenska University Hospital. The control PBMCs were isolated with dextran sedimentation followed by density gradient separation with Lymphoprep™ (Alere Technologies AS, #1114547). All cells were cryopreserved until usage.

### Flow cytometry

Stainings for extracellular antibodies were performed in phosphate buffered saline (PBS) with 0.5% BSA and 0.1% EDTA while stainings for intracellular antibodies were done in permeabilization buffer (eBioscience, #00-8333-56) following fixation and permeabilization of the cells (Fixation/Permeabilization Concentrate and Diluent; eBioscience, #00-5123-43, #00-5223-56). Cell viability was analyzed with LIVE/DEAD™ Fixable Yellow Dead Cell Stain Kit (Life Technologies, #L34959). A list of all conjugated antibodies can be found in Table S1. Flow cytometry analyses were conducted on a BD LSRFortessa™ instrument (BD Biosciences) with subsequent data analysis performed in BD FACSDiva Software version 8.0.1 (BD Biosciences). FACS gating strategy can be found in Fig. S1.

### Immunohistochemistry

Biopsies of metastases were obtained during the IHP procedure. The samples were fixed in neutral formaldehyde and embedded in paraffin. Four µM thick sections were mounted and used for immunohistochemical staining using standard immunoperoxidase protocols with magenta chromogen dye to better differentiate positive staining cells from brown (melanin) pigment. Antibodies against CD8 (clone: C8/144B, Dako, #IS623), CD68 (clone: KP1, Dako, #IR609), CD163 (clone: 10D6, Nordic BioSite, #LS-C87534) and PD-1 (clone: NAT105, Abcam, #ab52587) were applied after antigen retrieval, and the EnVision Flex high pH method was used on a Dako Autostainer Link 48.

Whole-slide-imaging at 40X magnification (Hamamatsu NanoZoomer S210) was used to capture the slides. Using viewing and image analysis software (Visiopharm Oncotopix) a circular area of 0.313 mm^2^ was superimposed on the peripheral part of the tumor metastasis (Fig. S2). Areas with necrosis, heavy pigmentation and larger vessels were excluded. The peripheral border area chosen to analyze was selected through CD8^+^ hotspot “eyeballing” in low power view. Thus, for each tumor, the border area showing the highest CD8^+^ T cell content was selected. This could be applied to 27/30 samples. Two samples (2/30) included only tumor tissue and one sample (1/30) had a sparse tumor mass (defined as a tumor less than three times the diameter of the superimposed circle’s diameter) why also the central part of the tumor was analyzed. For each biopsy, the same tumor area was used for analysis of all antibody stainings (i.e. CD8, CD68, CD163 and PD-1). All scanned immunohistochemistry slides were analyzed in a blinded fashion by one of the authors (JS). The image analysis software was adjusted to count only cells of a defined size and color intensity. The intensity was set to a level that included obviously positive cells and excluded unspecific staining. The size was set to exclude small spots that are not cells and other unspecific staining, including background staining.

### Statistical analyses

Statistical analyses were performed in GraphPad Prism 8.4.2 (GraphPad Software). All statistical tests were non-parametric; non-paired Mann-Whitney test, Spearman correlations and log-rank test for analysis of overall survival.

## Results

### Myeloid cells of patients with uveal melanoma liver metastasis are altered

**Figure 1.**
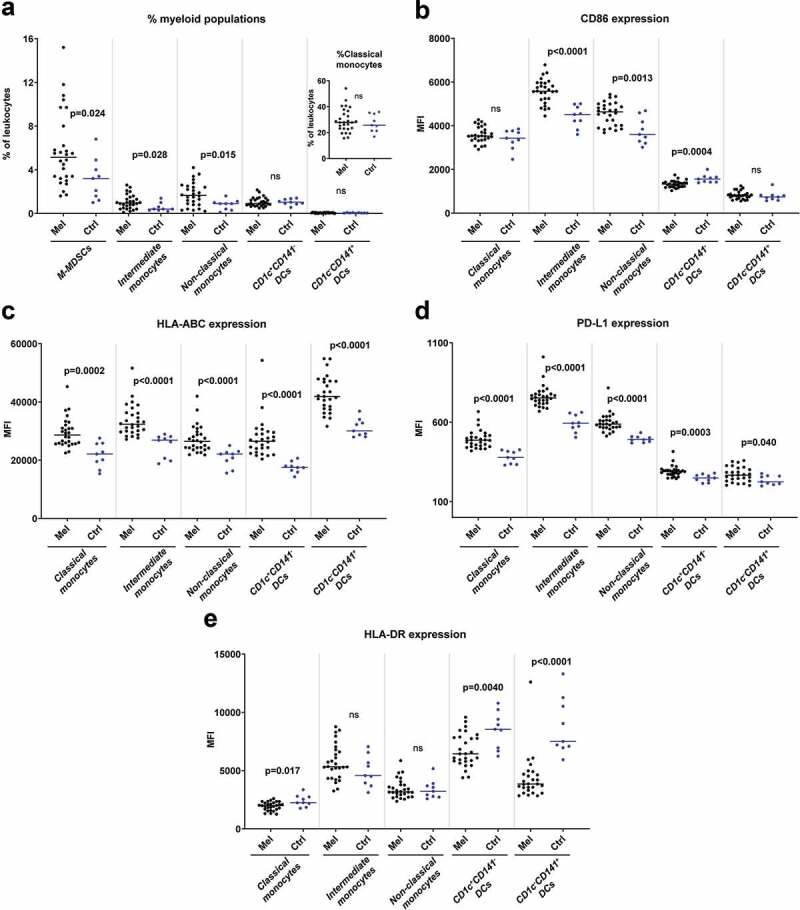
Myeloid cells in peripheral blood differ in uveal melanoma patients and in healthy controls. (a) The percentages of cells with a phenotype of M-MDSCs (CD33^+^CD14^+^HLA-DR^−/low^), classical monocytes (CD33^+^CD14^++^CD16^−^), intermediate monocytes (CD33^+^CD14^++^CD16^+^), non-classical monocytes (CD33^+^CD14^+^CD16^++^), CD1c^+^CD141^−^ DCs (CD33^+^CD14^−^CD16^−^HLA-DR^+^) and CD1c^−^CD141^+^ DCs among all leukocytes in peripheral blood for uveal melanoma patients (Mel) before IHP and for healthy controls (Ctrl). The expression of (b) CD86, (c) HLA-ABC, (d) PD-L1 and (e) HLA-DR on five of the myeloid populations (n_Melanoma_ = 28, n_Ctrl_ = 9, Mann-Whitney test). MFI = Median fluorescence intensity

Flow cytometry analyses of PBMCs showed differences in the myeloid compartments between uveal melanoma patients and healthy controls. The patient blood contained enhanced levels of CD14^+^HLA-DR^−^^/low^ cells, which is a phenotype typically associated with monocytic myeloid derived suppressor cells (M-MDSCs) (Figure 1a). Uveal melanoma patients also had higher fractions of CD16^+^ intermediate and non-classical monocytes which are regarded as inflammatory monocytes.^15^ The CD16^+^ monocytes in melanoma patients showed signs of activation as they expressed increased levels of CD86, HLA-ABC and PD-L1, compared with healthy controls (Figure 1b,d). Regarding dendritic cell (DC) populations, there were no significant differences in numbers for the CD1c^+^ DCs, which is the most frequent blood DC population, or for the CD141^+^ DCs that are specialized in cross-presenting antigens to CD8^+^ T cells.^16^ However, DCs from uveal melanoma patients expressed reduced levels of the antigen-presentation molecule HLA-DR and the costimulatory molecule CD86 (Figure 1b,e). Furthermore, all DC populations as well as the classical monocytes expressed enhanced levels of HLA-ABC and of PD-L1 (Figure 1c,d).

### T cells from patients with uveal melanoma show signs of immunosuppression

**Figure 2.**
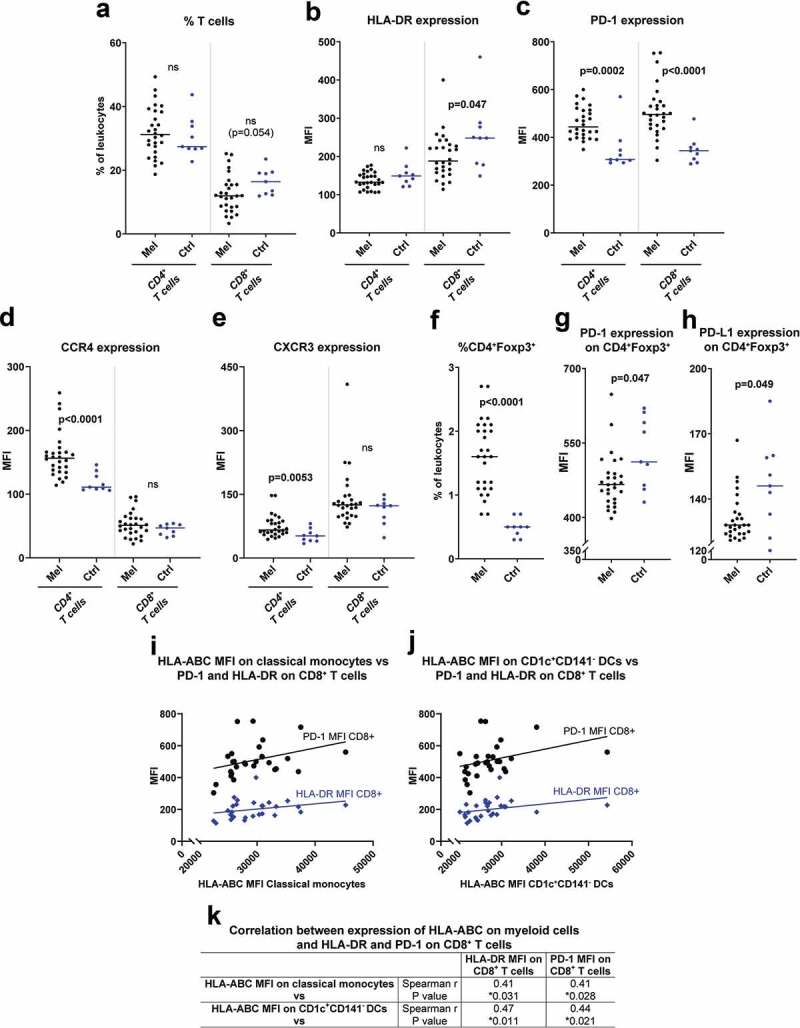
Presence of immunosuppressive T cell populations in peripheral blood of uveal melanoma patients. (a) The percentages of CD4^+^ and CD8^+^ T cells (CD3^+^) among all leukocytes in peripheral blood for uveal melanoma patients (Mel) before IHP and for healthy controls (Ctrl). The expression of (b) HLA-DR, (c) PD-1, (d) CCR4 and (e) CXCR3 on the T cell populations. (f) The percentage of CD4^+^Foxp3^+^ T cells among all leukocytes for patients and for controls, and the expression of (g) PD-1 and (h) PD-L1 on these cells (n_Melanoma_ = 28, n_Ctrl_ = 9, Mann-Whitney test). The expression of HLA-DR and PD-1 on CD8^+^ T cells is depicted against the expression level of HLA-ABC on (i) classical monocytes and of (j) CD1c^+^CD141^−^ DCs, with the lines adapted from linear regression. (k) The expression of HLA-ABC on classical monocytes and on CD1c^+^CD141^−^ DCs were correlated against the levels of HLA-DR and PD-1 on CD8^+^ T cells utilizing Spearman correlation (*n* = 28). MFI = Median fluorescence intensity

There was a trend toward a lower fraction of cytotoxic CD8^+^ T cells in peripheral blood of uveal melanoma patients compared with healthy controls (Figure 2a). The CD8^+^ T cells in patients expressed lower levels of HLA-DR (Figure 2b), an activation marker on T cells.^17^ The expression of PD-1, which is an inhibitory ligand for T cell stimulation that also may be upregulated during T cell activation, was higher on both CD4^+^ and CD8^+^ T cells from melanoma patients (Figure 2c). The CD4^+^ T cells in patients also showed higher expression levels of the chemokine receptors CCR4 and CXCR3 (Figure 2d,e), while there was no difference in CCR2 and CCR5 expression on CD4^+^ or CD8^+^ T cells (Fig. S3). The percentage of CD4^+^Foxp3^+^ T cells, which may be regarded as an immunosuppressive regulatory T cell (T_regs_) population, was much higher in patients than in healthy controls, while the expression of both PD-1 and PD-L1 on these cells was lower (Figure 2f,h).

Myeloid cells are responsible for antigen-presentation and activation of T cells. When correlating myeloid cell parameters and T cell parameters, it was found that patients with myeloid cells with high HLA-ABC expression also had CD8^+^ T cells expressing high levels of HLA-DR and PD-1 (Figure 2i,k). This may indicate that the T cells were activated by the HLA-ABC expressing myeloid cells, but further studies are needed to confirm this hypothesis.

### Activation of myeloid cells in peripheral blood in uveal melanoma patients correlates to the amount of CD8^+^ T cells in tumors

**Figure 3.**
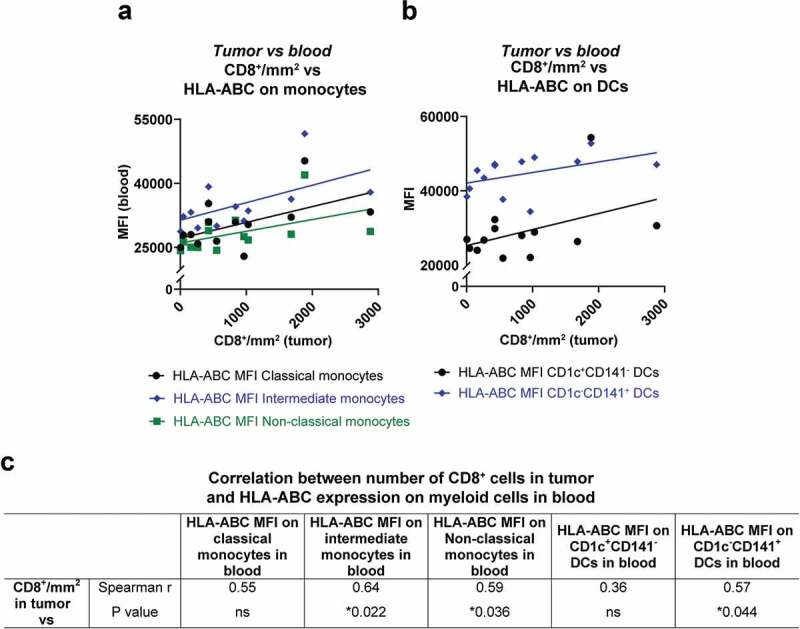
Correlation between activated myeloid cells in peripheral blood and CD8^+^ T cells in tumor. The number of CD8^+^ cells in tumor biopsies from uveal melanoma liver metastases are depicted against the expression of HLA-ABC on (a) monocytes and (b) on DCs in peripheral blood. All lines are adapted from linear regression. (c) The amount of CD8^+^ cells in tumor biopsies were correlated against the HLA-ABC expression on monocytes and on DCs, utilizing Spearman correlations (*n* = 13). MFI = Median fluorescence intensity

Tumor biopsies from liver metastases obtained prior to IHP were available from 13 of the patients for which blood samples had been analyzed. These sections were stained for e.g. CD8 and CD68 by immunohistochemistry. The amount of CD8^+^ cells in the tumor samples positively correlated with the HLA-ABC expression on CD16^+^ monocytes and on CD1c^−^CD141^+^ DCs in peripheral blood (Figure 3c,a), suggesting that the immune status of blood myeloid cells may be of relevance for generation of tumor infiltrating CD8^+^ T cells.

### Patients with a high infiltration of CD8^+^ and CD68^+^ cells in tumors have a longer overall survival

**Figure 4.**
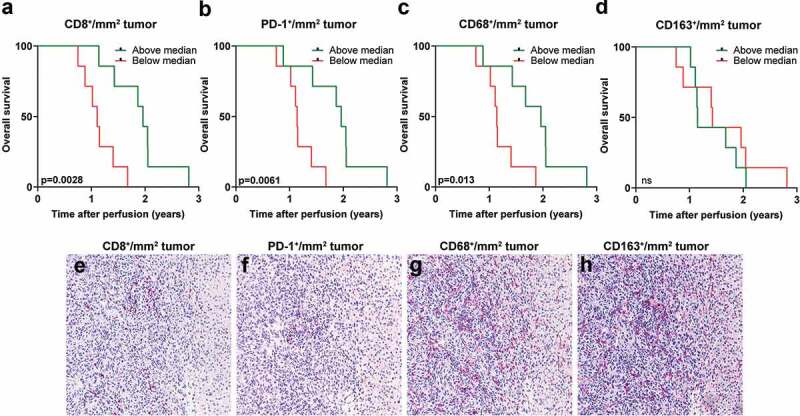
High tumor infiltration of CD8^+^ and CD68^+^ cells correspond to longer overall survival. A dichotomization of uveal melanoma patients based on above or below median infiltration of (a) CD8^+^ cells, (b) PD-1^+^ cells, (c) CD68^+^ and (d) CD163^+^ cells in liver metastases obtained prior to IHP correlated to overall survival (*n* = 14, log-rank test). Representative immunostainings from the same area in consecutively sectioned slides for (e) CD8, (f) PD-1, (g) CD68 and (h) CD163. Normal liver tissue is shown to the right part of each image

Biopsies from liver metastases obtained prior to IHP were available from a patient cohort where survival data were also available. Sections from these biopsies were analyzed for the presence of tumor-infiltrating CD8^+^, PD-1^+^, CD68^+^ and CD163^+^ cells (Figure 4e,h). When dichotomizing patients based on above or below median presence of CD8^+^ cells and analyzing patient survival within these two groups, it was clear that patients with a higher degree of tumor-infiltrating CD8^+^ cells had a longer overall survival, defined as time after treatment until death of any cause (Figure 4a). Similar analyses performed on PD-1^+^ and CD68^+^ tumor-infiltrating cells showed that patients with many PD-1^+^ and CD68^+^ cells had a longer overall survival after IHP (Figure 4b,c). In contrast, no survival benefit was seen with the presence of CD163^+^ cells (Figure 4d), which generally is regarded to be an immunosuppressive myeloid cell population^18^

### Effect of prior therapy on tumor infiltrating immune cells

**Figure 5.**
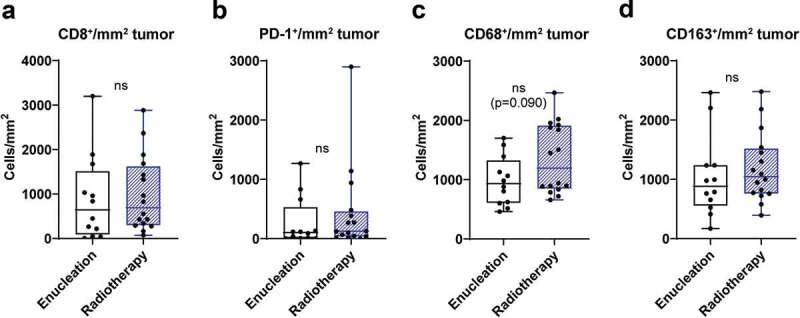
Tumor infiltration of immune cells and treatment of primary tumor. Uveal melanoma patients were dichotomized into two groups, enucleation or radiotherapy, based on treatment of their primary uveal melanoma. The number of tumor infiltrating (a) CD8^+^ cells, (b) PD-1^+^ cells, (c) CD68^+^ and (d) CD163^+^ cells in liver metastases obtained prior to IHP were thereafter analyzed in each group (*n* = 28, Mann-Whitney test)

The treatment the patients received for the primary tumor in the eye was enucleation or radiotherapy (see Table 1). To investigate the potential effect of primary treatment on immune cell profile in metastatic lesions, data from tumor infiltrating immune cells were pooled from both patient cohorts and compared between the two treatment groups. No significant differences were found when analyzing the content of tumor infiltrating CD8^+^, PD-1^+^, CD68^+^ and CD163^+^ cells (Figure 5a,d). However, there was a trend toward increased infiltration of CD68^+^ macrophages in patients that previously had received radiation therapy.

## Discussion

During recent years immunotherapy for the treatment of cutaneous malignant melanoma has been a success story with antibodies against CTLA-4 and PD-1 paving the way for a new era of immune checkpoint inhibitors. Despite the efficacy of these antibodies in the treatment of cutaneous melanoma, they have as of yet not been efficacious in the treatment of uveal melanoma. While the overall response rate for anti-PD-1 monotherapy is around 40% in cutaneous melanoma, it is only 4–5% in uveal melanoma.^6,7,19^ Despite originating from the same cell type, tumors from cutaneous and uveal melanoma show differences in their genetic profiles, which partly may explain the discrepancy in response rate toward immune checkpoint inhibitors. Tumors with a high mutational load are more likely to contain neo-antigens that T cells may recognize, and the mutational burden is known to be much higher in cutaneous melanoma than in uveal melanoma.^20^

In addition to a low mutational burden, the low immunogenicity and failed response to immunotherapy might have several other reasons. The two most important organs for uveal melanoma carcinogenesis, the eye where the primary tumors develop and the liver where the majority of the metastases occur, are organs with very distinct immune profiles. The eye is an immune privileged site with a restrictive immune system. In order to prevent inflammatory processes that may damage sight, the eye lacks a lymphatic system which limits the influx of immune cells. The eye is also protected by immunosuppressive agents, such as transforming growth factor β (TGF-β) and indoleamine 2,3-dioxygenase (IDO).^21,22^ The immune system of the liver is responsible for development of tolerance to food-derived antigens, and at the same time needs to protect the body against pathological antigens. Several of the cell types residing in the liver are immune cells or can directly affect the immune system, including Kupffer cells, liver sinusoidal endothelial cells (LSECs) and hepatic stellate cells (HSCs).^21,22^

Treatment of tumors with chemotherapeutic drugs might evoke an immune response due to the release of tumor-associated antigens from the dying tumor cells and the upregulated expression of danger-associated molecular patterns (DAMPs) on the surface of the cancer cells.^23,24^ Since earlier studies have shown that isolated regional perfusion of the limb with the chemotherapeutic drug melphalan for cutaneous melanoma is associated with a T cell response, ^10–13^ there is reason to believe the same might be true also for isolated regional perfusion of the liver. If this is the case, IHP might be one way to overcome the low immunogenicity of uveal melanoma and make this disease targetable for immunotherapies.

To this end, we characterized the phenotype of different immune cell populations in peripheral blood of uveal melanoma patients with liver metastases prior to IHP and performed comparisons to healthy controls. Uveal melanoma patients harbored a higher fraction of cells with an M-MDSC phenotype (CD14^+^HLA-DR^−^^/low^). M-MDSCs have been shown to be enriched in blood and tumors in many cancer patients, including cutaneous melanoma, where they suppress T cell responses.^25^ Uveal melanoma patients also harbored enhanced levels of CD16^+^ monocytes (intermediate and non-classical monocytes), which showed signs of enhanced activation as reflected by a higher expression of CD86, HLA-ABC and PD-L1. CD16^+^ monocytes accumulate during inflammatory conditions and produce higher levels of pro-inflammatory cytokines, such as TNF-α and IL-1β, compared with classical monocytes.^15^ Interestingly, while uveal melanoma patients showed higher expressions of MHC class I (HLA-ABC) on all investigated myeloid populations, the levels of MHC class II (HLA-DR) were lower on classical monocytes and on both analyzed DC populations. This might indicate that these cells have an impaired antigen-presenting capability. In addition, CD1c^+^ DCs also showed reduced expression of CD86, further indicating an impaired T cell activating function. Furthermore, all analyzed myeloid populations expressed enhanced levels of the inhibitory receptor PD-L1. Enhanced expression of PD-L1 on myeloid cells has previously been observed in cutaneous metastatic melanoma.^26^ Taken together, the net effect of the observed alteration in myeloid cells in peripheral blood of patients with uveal melanoma appears to be toward immunosuppression and reduced T cell priming.

Though there were no significant differences in the percentage of CD4^+^ and CD8^+^ T cells between uveal melanoma patients and healthy blood donors, patients tended to have a lower fraction of cytotoxic CD8^+^ T cells and a higher fraction of helper CD4^+^ T cells, in particular of a regulatory T cell (T_reg_) phenotype as indicated by an abundance of CD4^+^ T cells with a high expression of Foxp3. T_regs_ accumulate in several forms of cancer, and suppress antigen-presentation and T cell activation by various mechanisms.^27^ Further characterization of the T cell populations showed that CD8^+^ T cells had reduced expression of the activation marker HLA-DR. In addition, the expression of the inhibitory receptor PD-1 was higher in blood in patients compared with healthy controls on both CD4^+^ and CD8^+^ T cells. In addition to exerting T cell suppressive functions, PD-1 may be regarded as a marker for activation since it is upregulated on T cells during stimulation through the T cell receptor.^28^ The levels of HLA-ABC on myeloid cells correlated both to the expression of PD-1 and HLA-DR on T cells, suggesting that these markers indeed may be regarded as activation markers.

We had access to both blood samples and tumor biopsies from liver metastases obtained prior to IHP for some of the patients. Correlation analyses showed that the number of CD8^+^ T cells in tumor tissue positively correlated with the expression of HLA-ABC on myeloid cell populations in peripheral blood, which might indicate that the peripheral blood myeloid cell compartment is involved in shaping the tumor microenvironment. However, it was not possible to perform deeper analyses of the observed correlations, which thus are speculative and need to be confirmed in further studies.

In order to investigate the clinical relevance of immune cell populations in uveal melanoma, we analyzed the amount of tumor-infiltrating immune cells in biopsies from liver metastases in a patient cohort where survival data were available. It was clear that patients with a high degree of infiltrating CD8^+^ T cells had a longer overall survival. Moreover, a high infiltration of PD-1^+^ cells also correlated to a longer overall survival, suggesting that this was a sign of the presence of activated T cells. However, it was not possible to determine which lymphocyte population that expressed PD-1. It was also favorable for the patients to have a high amount of tumor-infiltrating CD68^+^ macrophages. Since CD68 is considered a marker for all types of macrophages, we also analyzed the amount of CD163^+^ cells in the sample material. CD163 is considered a marker for the more pro-tumorigenic M2 macrophage.^18^ No connection could be found between high amounts of CD163^+^ cells and survival, suggesting that it was the presence of CD68^+^CD163^−^ M1 stimulatory macrophages that correlated to a favorable IHP response.

Approximately half of the patients in the two cohorts received radiotherapy as treatment of their primary disease. Radiotherapy may cause immunogenic cell death in cancer cells in a similar way as certain chemotherapeutic drugs.^29^ Although there were no significant differences in immune profiles of metastatic tumors arising in radiotherapy treated patients compared with patients treated by enucleation, there was a trend toward a higher infiltration of macrophages in patients treated by radiotherapy. Further studies are needed to elucidate if there is a true relation between radiotherapy and immune activation in uveal melanoma patients.

The image analysis software used for the quantification analysis proved to have certain limitations such as problems with identifying lowly positive labeled cells (particularly for the cytoplasmic and/or membranous stains) which could lead to a lower absolute count of positively stained cells. However, this issue applied to all the biopsies in the specific stain and would therefore not affect the significant outcome.

The sample material we had access to was rather small due to the rarity of metastatic uveal melanoma. This is a limitation of this study since it impedes the drawing of generalized conclusions. The survival analyses would have benefitted from the inclusion of clinical variables in a multivariate setting, but unfortunately this was not possible due to the small sample size.

In conclusion, patients with uveal melanoma liver metastases harbor increased fractions of immunosuppressive and inflammatory immune cells, while DCs and CD8^+^ T cells show signs of impaired activation, compared to healthy controls. Nevertheless, the survival analysis suggests that patients with sufficient levels of tumor-infiltrating CD8^+^ T cells and non-M2 macrophages have an increased survival following IHP. This implies that the efficacy of IHP with melphalan to some extent may rely on the immune system, and that further studies investigating IHP combined with immunotherapy are warranted.

## Supplementary Material

Supplemental Material

Supplemental Material
