# Supplementary material for: Presence of tumor-infiltrating CD8+ T cells and macrophages correlates to longer overall survival in patients undergoing isolated hepatic perfusion for uveal melanoma liver metastasis
Source: Oncoimmunology. 2020 Dec 10;9(1):1854519. doi: 10.1080/2162402X.2020.1854519 (PMC7733984; doi:10.1080/2162402X.2020.1854519)
Supplement: Supplemental Material [file KONI_A_1854519_SM1377.docx]

**SUPPLEMENTARY MATERIALS**

| **Antigen** | **Fluorophore** | **Clone** | **Vendor** | **Article number** |
| --- | --- | --- | --- | --- |
| CCR2 | PE | REA624 | Miltenyi Biotec | 130-109-595 |
| CCR4 | BV421 | 1G1 | BD Biosciences | 562579 |
| CCR5 | FITC | 2D7 | BD Biosciences | 561747 |
| CD14 | APC-Cy7 | MφP-9 | BD Biosciences | 333951 |
| CD141 | APC | 1A4 | BD Biosciences | 564123 |
| CD16 | BV605 | 3G8 | BD Biosciences | 563172 |
| CD1c | BV421 | L161 | BioLegend | 331526 |
| CD3 | BV711 | UCHT1 | BD Biosciences | 563725 |
| CD3 | FITC | HIT3a | BD Biosciences | 555339 |
| CD33 | PE-Cy7 | P67.6 | BD Biosciences | 333952 |
| CD4 | APC-H7 | RPA-T4 | BD Biosciences | 560158 |
| CD45 | BV786 | HI30 | BD Biosciences | 563716 |
| CD8 | PerCP-Cy5.5 | RPA-T8 | BD Biosciences | 560662 |
| CD86 | BV711 | 2331 | BD Biosciences | 563158 |
| CXCR3 | BUV395 | 1C6 | BD Biosciences | 565223 |
| Foxp3 | PE | 3G3 | Miltenyi Biotec | 130-093-014 |
| HLA-ABC | FITC | G46-2.6 | BD Biosciences | 555552 |
| HLA-DR | FITC | L243 | BD Biosciences | 347400 |
| HLA-DR | PerCP | L243 | BD Biosciences | 347402 |
| PD-1 | BV421 | EH12.1 | BD Biosciences | 562516 |
| PD-L1 | BUV395 | MIH1 | BD Biosciences | 740320 |
| PD-L1 | PE-Cy7 | MIH1 | eBioscience | 25-5983-73 |

**Table S1**. List of all conjugated antibodies for flow cytometry utilized in the study.
