# Supplementary material for: Presence of tumor-infiltrating CD8+ T cells and macrophages correlates to longer overall survival in patients undergoing isolated hepatic perfusion for uveal melanoma liver metastasis
Source: Oncoimmunology. 2020 Dec 10;9(1):1854519. doi: 10.1080/2162402X.2020.1854519 (PMC7733984; doi:10.1080/2162402X.2020.1854519)

**SUPPLEMENTARY FIGURES**


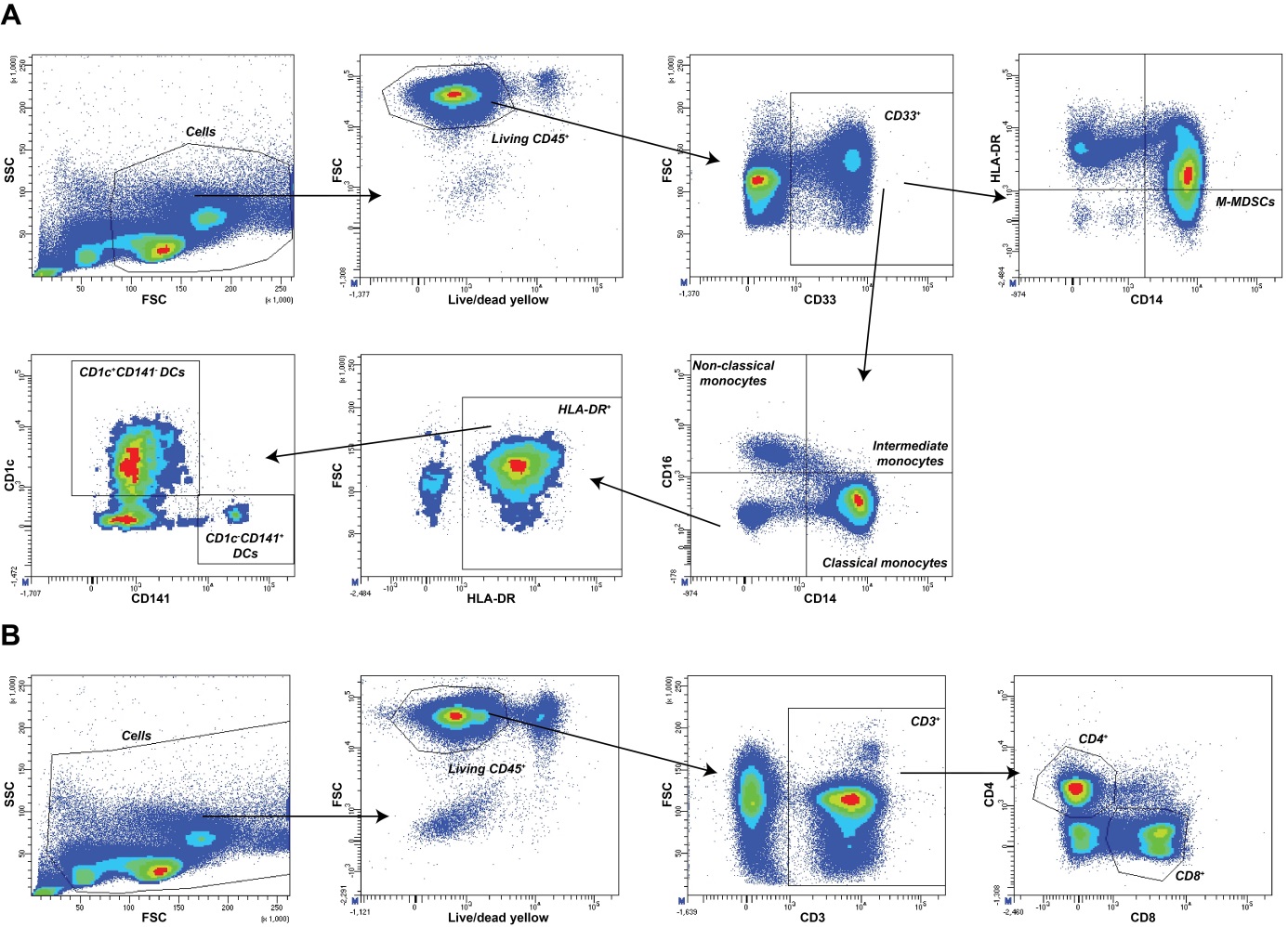


**Figure S1**. FACS gating strategy for **A)** myeloid cells and **B)** T cells.


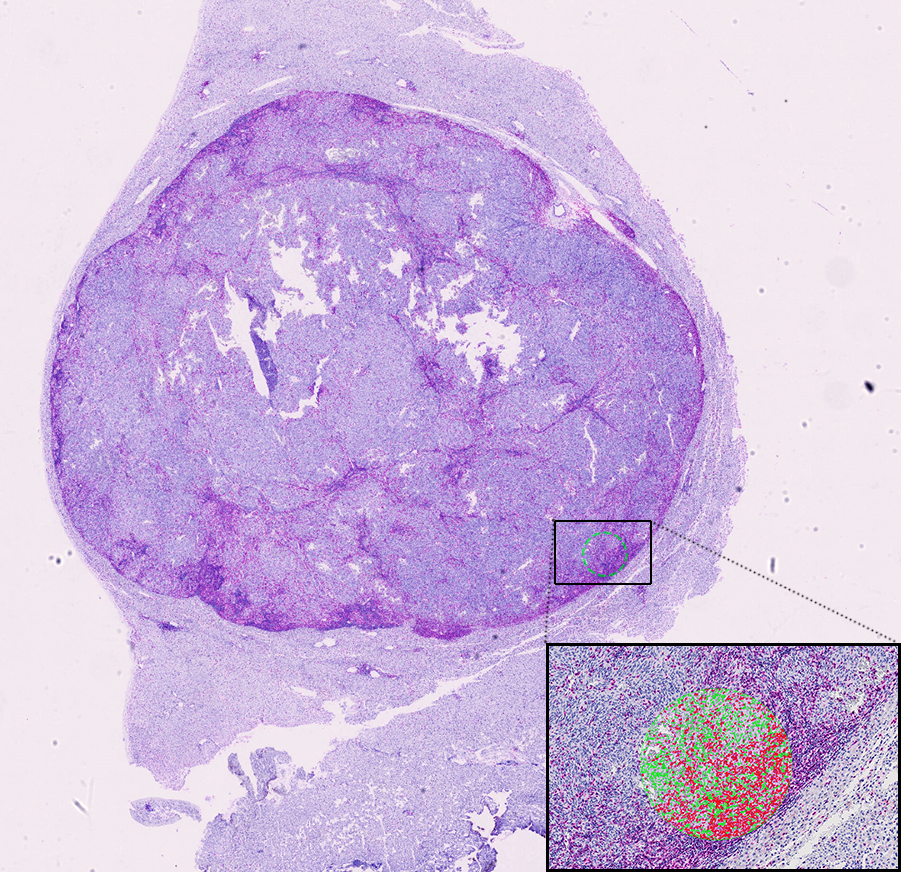


**Figure S2***.* Low magnification overview of a uveal melanoma liver metastasis (diameter 9.5 mm). The inflammatory infiltrate is most pronounced in the tumor periphery. The insert shows where the measurement was done (inside the green circle). The magenta immunohistochemically stained cells are CD8^+^ T cells and green cells are negative cells, mainly lymphocytes.

**Figure S3**. The expression of **A)** CCR2 and **B)** CCR5 on CD4^+^ and CD8^+^ T cells in peripheral blood for uveal melanoma patients (Mel) before IHP and for healthy controls (Ctrl) (n_Melanoma_=28, n_Ctrl_=9, Mann-Whitney test). MFI = Median Fluorescence Intensity.


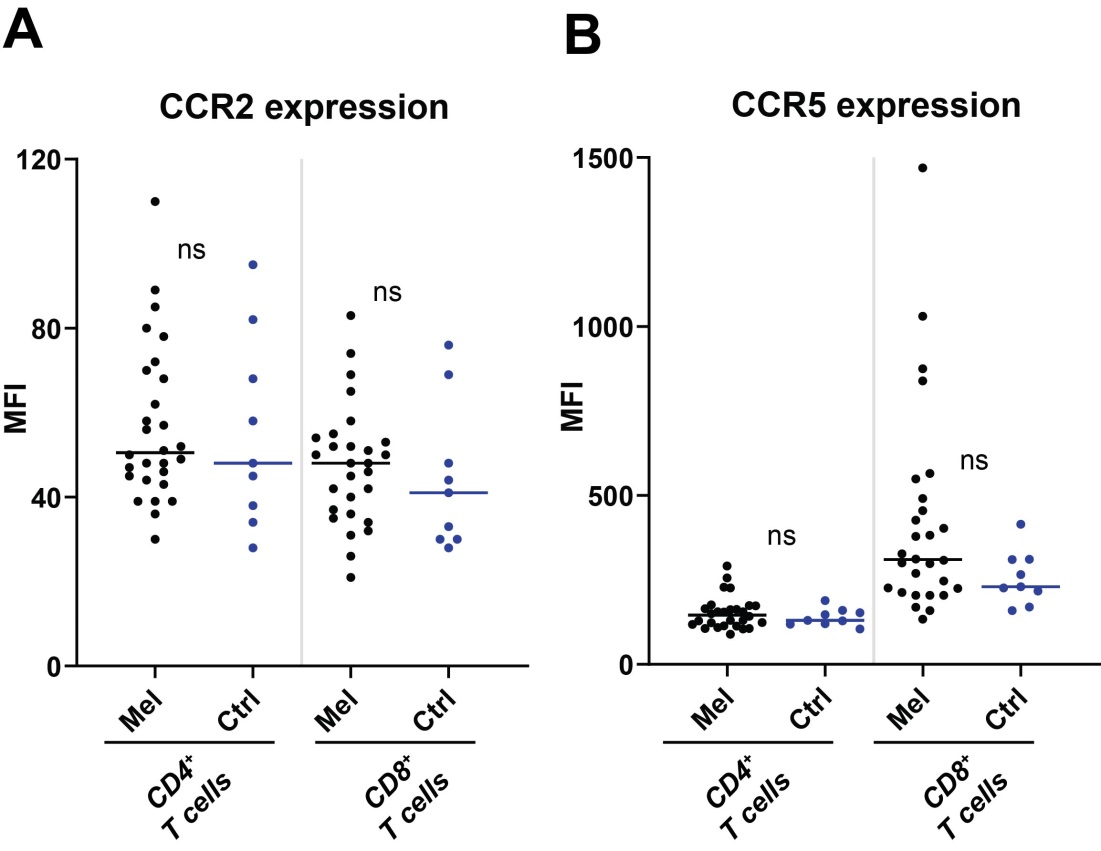

Supplement: Supplemental Material [file KONI_A_1854519_SM1376.docx]
